# ZIF-67 Derived Co_2_VO_4_ Hollow Nanocubes for High Performance Asymmetric Supercapacitors

**DOI:** 10.3390/nano12050848

**Published:** 2022-03-02

**Authors:** Chengda Li, Dongliang Ma, Qinglin Zhu

**Affiliations:** 1 Xinjiang Key Laboratory of Solid State Physics and Devices, Xinjiang University, Urumqi 830017, China; lichengda572@163.com (C.L.); linzyznil@163.com (Q.Z.); 2 School of Physical Science and Technology, Xinjiang University, Urumqi 830017, China

**Keywords:** supercapacitor, zeolitic imidazolate frameworks, Co_2_VO_4_ nanocubes, hollow structures

## Abstract

In this work, a new type of Co_2_VO_4_ hollow nanocube (CoVO-HNC) was synthesized through an ion exchange process using ZIF-67 nanocubes as a template. The hollow nanocubic structure of the CoVO-HNC provides an abundance of redox sites and shortens the ion/electron diffusion path. As the electrode material of supercapacitors, the specific capacitance of CoVO-HNC is 427.64 F g^−1^ at 1.0 A g^−1^. Furthermore, an asymmetric supercapacitor (ASC) was assembled using CoVO-HNC and activated carbon (AC) as electrodes. The ASC device attains an energy density of 25.28 Wh kg^−1^ at a high-power density of 801.24 W kg^−1^, with 78% capacitance retention after 10,000 cycles at 10 A g^−1^.

## 1. Introduction

As electronic devices are updated in today’s society, the demand for efficient energy storage devices is increasing dramatically. Supercapacitors, also known as electrochemical capacitors, have attracted great interest from researchers in recent years due to their environmental friendliness, fast charge and discharge process, long cycle life, and high-power density [1,2,3]. However, the low energy density limits their practical application [4]. Electrode materials are one of the key factors that determine the electrochemical performance of supercapacitors [5]. The development of new supercapacitors electrode materials to improve the energy density of supercapacitors has become a research hotspot. Studies have shown that ternary metal oxides exhibit excellent electrochemical performance when used as supercapacitor electrode materials through the redox reaction between two different metal cations and the synergy between them [6,7,8]. As a transition metal element, vanadium has the characteristics of multivalent state (+2–+5) and easily deformable V-O polyhedron, which in turn produces a large amount of vanadium oxide compounds (M*_x_*V*_y_*O*_z_*) [9,10]. The introduction of different types of metal ions M to M*_x_*V*_y_*O*_z_* can show different electrochemical properties, which provides a large number of options for the study of supercapacitors electrode materials [11]. For example, Butt et al. reported a solvothermal method to prepare layered nanospheres of ZnV_2_O_4_. Electrochemical test results show that the specific capacitance is 360 F g^−1^ when the current density is 1.0 A g^−1^. After 1000 cycles, the capacitance retention rate can reach 89%, with excellent stability [12]. Sun et al. prepared 3D Co_2_V_2_O_7_·3.3H_2_O micro-flower by a co-sedimentation technique. Due to the micro-flower structure and porosity, the specific capacitance can reach 351 F g^−1^ at 1 A g^−1^ when used as an electrode material for supercapacitors [13]. Liu et al. successfully prepared orderly stacked CoV_2_O_6_·2H_2_O nanosheets with a microwave-assisted method, and its energy density reached 19 Wh kg^−1^ at the power density 400 kW kg^−1^ [14]. Nithya et al. prepared FeVO_4_ nanoparticles, when combined with LiCoPO_4_ as a positive electrode to form ASC, provided an excellent energy density of 21 Wh kg^−1^ at a power density of 1326 W kg^−1^ [15].

Porous metal-organic frameworks (MOFs) are polymers composed of organic ligands and metal ions through coordination bonds. With properties such as large specific surface area, large pore volume, and controlled pore size and structure, they are widely used in energy storage, electrocatalysis, sensors, drug release, and other fields [16]. Zeolitic imidazole frameworks (ZIFs), a typical material for MOFs, are ideal templates due to their high thermal and chemical stability [17]. The electrode materials of supercapacitors made with ZIFs as templates can inherit the characteristics of MOFs materials. The large specific surface area provides more electroactive sites, facilitating the contact between the electrode material and electrolyte ions. At the same time, the porosity can provide transport pathways for ion/electron diffusion [18]. In addition, the resulting hollow structure can effectively alleviate the problems of volume expansion and structural change caused by the electrode material during the charging and discharging process [19,20]. ZIFs-derived composites have made very exciting progress as electrode materials for supercapacitors. For example, Kisan Chhetri et al. prepared Co_3_O_4_-PANI@ZIF-8NPC nanocomposites by controlling the in-situ polymerization of aniline and Co_3_O_4_NFs on the surface of ZIF-8NPC. The ACS assembled with Co_3_O_4_-PANI@ZIF-8NPC and ZIF-8NPC exhibited a high capacitance retention rate of 88.43% after 10,000 cycles at a current density of 10Ag^−1^. In addition, at a power density of 751.51 W kg^−1^, the energy density was as high as 52.81 Wh kg^−1^ [21]. Such excellent electrochemical performance provides a new choice for supercapacitor electrode materials. With the development of wearable electronic devices, flexible supercapacitors have become a research hotspot in recent years. Kisan Chhetri et al. transformed ACFT of grown 2D Co-MOF arrays into Se_x_@CPNA-ACFT through successive phosphidization and selenium infiltration processes. The flexible solid-state ACS assembled with Se_x_@CPNA-ACFT and FeS_2_@rGO-ECFT exhibited high capacitance retention of 92.4% after 10,000 cycles at a current density of 150 mA cm^−2^. Notably, at a power density of 0.335 kW kg^−1^, the energy density was as high as 0.335 kW kg^−1^ [22].

As a result, supercapacitors electrode materials prepared from ZIFs as templates exhibit excellent electrochemical performance. In this study, we combined the advantages of vanadium oxide and MOFs materials to prepare Co_2_VO_4_ hollow nanocubes (CoVO-HNC) to obtain excellent electrochemical performance. Firstly, using previous reports, we prepared ZIF-67 nanocubes as template [23]. Then, ZIF-67 was transformed into CoVO-HNC by solvothermal method using NH_4_VO_3_ and a small amount of ammonia water. After the three-electrode system test, the CoVO-HNC electrode material exhibits a specific capacitance of 427.64 F g^−1^ at 1 A g^−1^. Furthermore, an ASC was constructed using AC as the negative electrode and CoVO-HNC as the positive electrode. The device showed a large energy density of 25.28 Wh kg^−1^ at a power density of 801.24 W kg^−1^. These results indicate that the synthesized CoVO-HNC has considerable application prospects as electrode materials for supercapacitors.

## 2. Materials and Methods

### 2.1. Materials and Chemical Reagents

All chemicals are analytical grade and used directly without any purification. The Co (NO_3_)_2_·6H_2_O, NH_4_VO_3_, NH_3_·H_2_O, 2-Methylimidazole (2-MeIm), cetyltrimethylammonium bromide (CTAB), ethanolamine, and absolute ethanol were offered by Aladdin Chemical Co., Ltd., Shanghai, China.

### 2.2. Synthesis of ZIF-67 Nanocubes

ZIF-67 was synthesized according to previous literature [23]. First, 15 mg of CTAB was dissolved in 30 mL of deionized water. Subsequently, 874 mg of Co (NO_3_)_2_·6H_2_O was added to form a pink solution. The above pink solution was added quickly into 60 mL of aqueous solution containing 5.76 g of 2-MeIm and stirred at room temperature for 1 h. Lastly, the purple precipitate was collected by centrifugation and washed with absolute ethanol.

### 2.3. Synthesis of CoVO-HNC

**Scheme 1:**
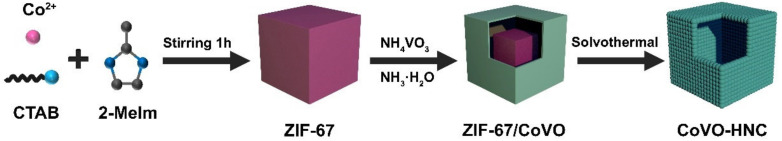
Illustration of the fabrication process of CoVO-HNC.

Typically, 50 mg of the synthesized ZIF-67 was dispersed into 40 mL absolute ethanol under ultrasonication to obtain a homogeneous dispersion. At the same time, 46.79 mg NH_4_VO_3_ and 0.5 mL NH_3_·H_2_O were dissolved in 9.5 mL deionized water. Then, the NH_4_VO_3_ aqueous solution was added to the ZIF-67 solution with continuous stirring for 10 min. The precipitate collected by centrifugation was dispersed in 40 mL of absolute ethanol. Lastly, the suspension in a stainless-steel autoclave was heated at 120 °C for 4 h. After natural cooling, samples were collected by centrifugation and washed several times. The sample was dried at 60 °C for 8 h to obtain the CoVO-HNC (see Scheme 1).

### 2.4. Synthesis of Co_3_O_4_ Hollow Nanocubes (CoO-HNC)

In order to obtain the CoO-HNC, the as-synthesized ZIF-67 was annealed at 350 °C for 2 h in a vacuum.

### 2.5. Synthesis of Co_2_VO_4_ Nanoparticles (CoVO-NP)

First, 0.117 g NH_4_VO_3_ was added to 50 mL of deionized water and heated to 80 °C with stirring for 1 h to dissolve. Subsequently, 0.582 g of Co (NO_3_)_2_·6H_2_O was added to the above solution, and the pH value of the solution was adjusted to 9.0 with ethanolamine. The obtained mixture was transferred to 100 mL Teflon-lined stainless-steel autoclave, which was maintained at 150 °C for 10 h. After cooling to ambient temperature naturally, the resultant precipitate was washed with deionized water and absolute ethanol, and then dried at 60 °C for 8 h. Finally, the prepared precipitate was calcined at 350 °C for 2 h in a vacuum environment.

### 2.6. Material Characterization

The as-prepared samples were subjected to crystallographic studies by X-ray diffraction (XRD, Bruker D8 Advance, Bruker-AXS, Karlsruhe, Germany). The morphology and microstructure of the samples were analyzed by field-emission scanning electron microscopy (FE-SEM, S-4800, Hitachi, Tokyo, Japan) and transmission electron microscopy (TEM, Tecnai G2 F20 U-TWIN, FEI Company, Hillsboro, OR, USA). Elemental compositions and surface valences were investigated using X-ray photoelectron spectroscopy (XPS, ESCALAB 250Xi, Thermo-Fisher Scientific, Waltham, MA, USA). The specific surface area and pore size distribution were computed using the Brunauer–Emmett–Teller (BET) and Barrett–Joyner–Halenda (BJH) from the N_2_ adsorption-desorption isotherm (Micromeritics ASAP 2460, Micromeritics, Atlanta, GA, USA).

### 2.7. Electrochemical Characterization

The active material (80 wt%), carbon black (10 wt%), and polyvinylidene fluoride (10 wt%) were mixed into a slurry and uniformly coated on nickel foam (1 cm^2^) to prepare the working electrode. The as-prepared working electrode, a platinum foil counter electrode, and Hg/HgO reference electrode were assembled into a three-electrode system in 3 M KOH electrolyte. Electrochemical performance tests were performed on an electrochemical workstation (Zennium IM6, ZAHNER, Kronach, Germany). Test contents include cyclic voltammetry (CV), galvanostatic charge-discharge (GCD), and electrochemical impedance spectroscopy (EIS, 100 K−0.01 Hz).

The specific capacitance (F g^−1^) was calculated from the GCD curve as the following equation [24]:

(1) $$C=\frac{i\Delta t}{m\Delta V}\;$$

where *i* (A), Δ*t* (s), *m* (mg), and ΔV (V) are discharge current, discharge time, mass of the active material, and potential window, respectively.

An ASC was fabricated by CoVO-HNC and AC as positive electrode and negative electrode with 3 M KOH electrolyte. The loading mass of the CoVO-HNC and AC electrode materials was determined according to the following equation [25]:

(2) $$\frac{m^{+}}{m^{-}}=\frac{C^{-}\Delta V^{-}}{C^{+}\Delta V^{+}}\;$$

where *m* (mg), *C* (F g^−1^), and ΔV (V) represent loading mass, specific capacitance, and potential window of positive (+) and negative (−) electrodes, respectively.

The energy density (E, Wh kg^−1^) and power density (P, W kg^−1^) were determined according to the following equations [26]:

(3) $$E=\frac{C\Delta V^{2}}{2\times 3.6}\;$$

(4) $$P=\frac{3600\times E}{\Delta t}\;$$

where C (F g^−1^), ΔV (V), and Δt stand for the specific capacitance of ASC device, potential window, and discharge time, respectively.

## 3. Results

### 3.1. Morphology and Structure Characterization

**Figure 1:**
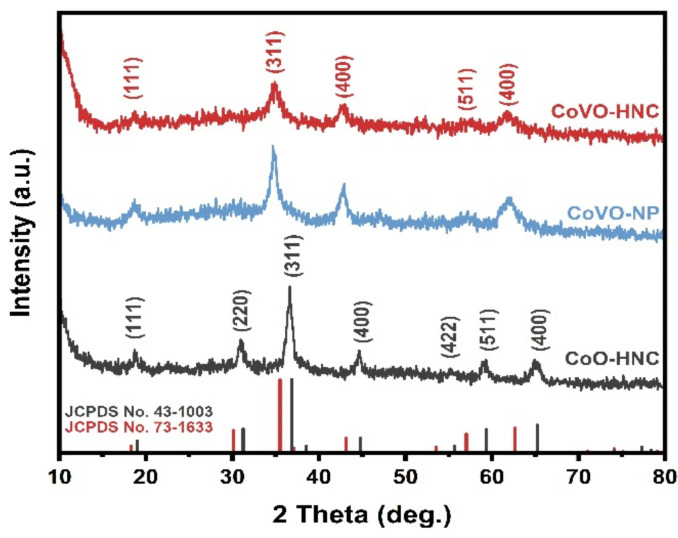
XRD pattern of CoO-HNC, CoVO-NP, and CoVO-HNC.

The prepared samples were characterized by XRD. As well displayed in Figure S1 (Supplementary Materials), the diffraction peaks of the prepared ZIF-67 template are sharp and narrow, indicating that the material has high crystallinity. The diffraction peak position is consistent with the simulated XRD pattern, which proves that ZIF-67 has been successfully prepared. Figure 1 shows the XRD patterns of the CoO-HNC, CoVO-NP, and CoVO-HNC. It can be seen that the diffraction peaks of CoVO-HNC and CoVO-NP at 2θ = 18.3°, 35.5°, 43.1°, 57.0°, and 62.6° can be well attributed to the (111), (311), (400), (511), and (440) crystallographic planes of the cubic phase Co_2_VO_4_ (JCPDS #73-1633). According to the intensity contrast of diffraction peaks, the crystallinity of CoVO-HNC is slightly lower than that of the CoVO-NP. In addition, all diffraction peaks of CoO-HN correspond well to the cubic phase Co_3_O_4_ (JCPDS#74-1656). This means that CoVO-HNC and CoO-HNC were successfully derived from ZIF-67, and CoVO-NP was prepared with high purity.

**Figure 2:**
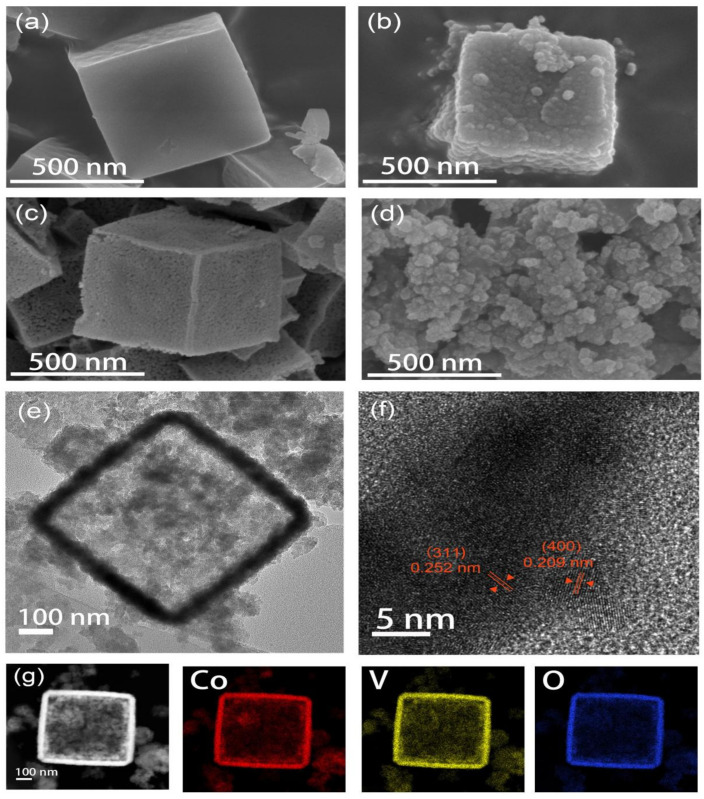
FESEM images of (**a**) ZIF-67, (**b**) CoVO-HNC, (**c**) CoO-HNC, and (**d**) CoVO-NP; (**e**,**f**) HRTEM image and (**g**) EDX mapping of CoVO-HNC.

The morphology and microstructure of the samples were characterized using FESEM and HRTEM. As shown in Figure 2a, the prepared ZIF-67 template has a cubic structure and the average particle size is 560 nm. The formation of the cubic structure is due to the addition of CTAB during the preparation process that inhibits the growth of {100} facets and accelerates the growth of {110} facets [27]. More importantly, the surface of the cube is smooth and the edge is clear, indicating that the synthesized ZIF-67 has higher purity [28]. The CoVO-HNC prepared with ZIF-67 as the template is shown in Figure 2b, which can be seen to maintain the cubic structure of the template. Furthermore, the rough surface of CoVO-HNC is caused by structural changes during the ion exchange process. The rough surface exposes more active sites, which enhances the capacitive properties [29]. Figure 2c shows that the CoO-HNC prepared by the template method also maintains a cubic structure with a rough and porous surface and a hollow interior. The CoVO-NP prepared by the template-free method presents a nanoparticle morphology as shown in Figure 2d. The hollow structure inside CoVO-HNC can be seen in the TEM image of Figure 2e. However, the hollow structure stems from the fact that the cobalt ion diffusion rate is faster than that of metavanadate ions [30], forming a void between ZIF-67 and the Co_2_VO_4_ shell. In this process, the ZIF-67/CoVO core-shell structure was formed. Subsequently, the internal ZIF-67 was completely consumed, and ZIF-67/CoVO was converted to CoVO-HNC. Furthermore, the hollow structure can alleviate the volume expansion during charging and discharging to prevent structural damage, thereby prolonging the service life [31]. HRTEM images (Figure 2f), distinct lattice stripes of CoVO-HNC can be observed with crystal plane spacing of 0.252 nm and 0.209 nm, corresponding to the (311) and (400) crystal planes, in agreement with the XRD results. Figure 2g shows EDS mapping of a single CoVO-HNC revealing the uniform distribution of cobalt, vanadium, and oxygen. As shown in Figure S2a,b, confirmed by Brunauer–Emmett–Teller, the specific surface areas of CoVO-HNC and CoVO-NP reached 52.6226 m^2^ g^−1^ and 29.5887 m^2^ g^−1^, respectively. The large specific surface area of the CoVO-HNC is beneficial to provide more active sites for the redox reaction. Typical IV type isotherms indicate that they are mesoporous materials. According to the Barrett–Joyner–Halenda test (illustration in Figure S2), the average pore diameters of CoVO-HNC and CoVO-NP are 19.5 nm and 21.7 nm, respectively. This mesoporous structure facilitates the diffusion of electrons and ions.

**Figure 3:**
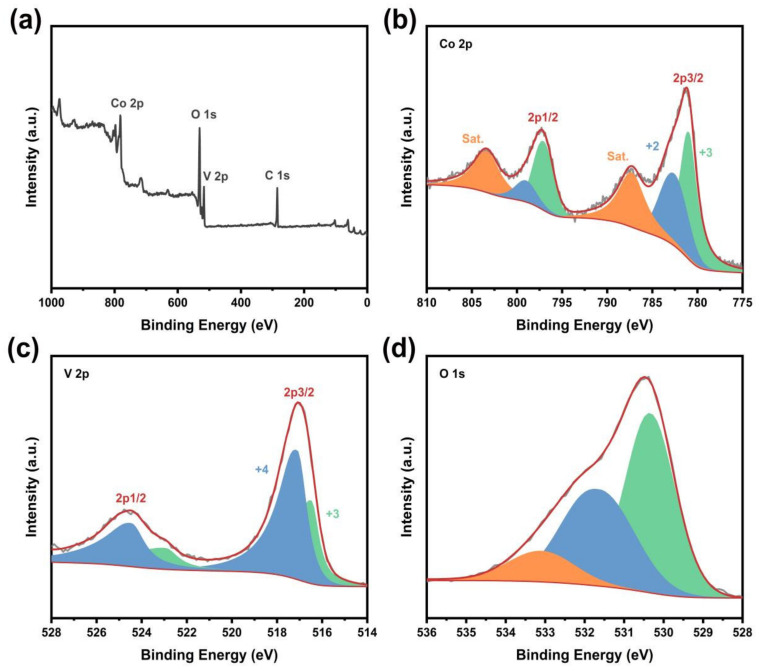
XPS spectra of CoVO-HNC (**a**) full spectrum, (**b**) Co 2p, (**c**) V 2p, and (**d**) O 1s spectra.

The element composition and chemical valence state of CoVO-HNC was studied by X-ray photoelectron spectroscopy (XPS). According to the survey spectrum (Figure 3a), CoVO-HNC was found to contain Co, O, V, and C elements, which is consistent with the results of EDS analysis. The C element may come from absorbed CO_2_ [32]. As shown in Figure 3b, the Co 2p spectrum is composed of two satellite peaks (denoted by “Sat.”) and two spin-orbit doublets (Co 2p_3/2_ and Co 2p_1/2_). The peaks located at 780.8 and 796.6 eV indicating the existence of Co^3+^, as well those at 782.3 and 798.1 eV, correspond to Co^2+^ [33]. In Figure 3c, the V 2p spectrum shows the presence of two oxidation states at 516.6 and 517.1 eV, which is consistent with the previous reports [34]. As shown in Figure 3d, the O 1s spectrum is located at the three fitting peaks of 530.4, 531.7, and 533.1 eV, which are attributed to the metal-oxygen bonds, surface absorbed water molecule, and oxygen ions in low coordination, respectively [35].

### 3.2. Electrochemical Performance

**Figure 4:**
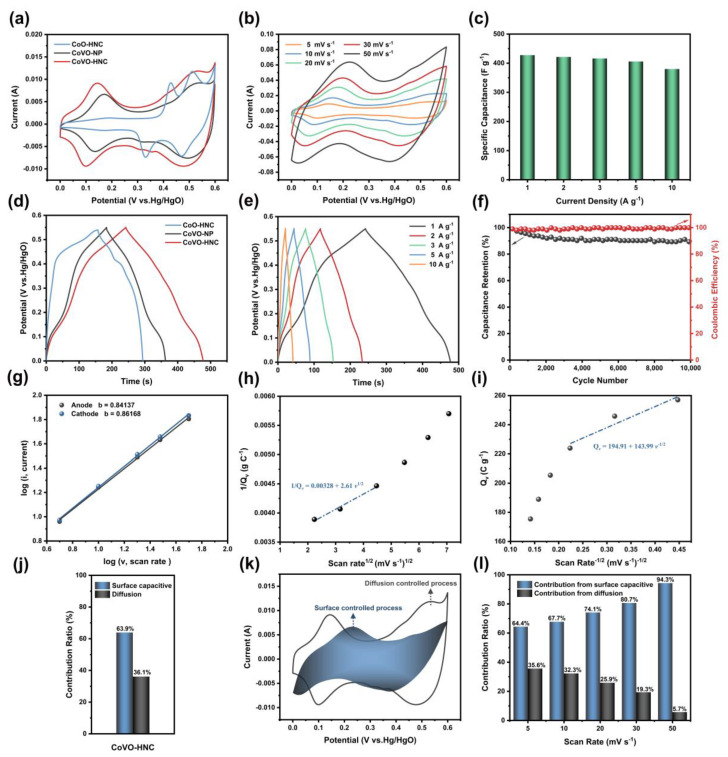
(**a**) CV curves of CoO-HNC, CoVO-HNC, and CoVO-NP at 5 mV s^−1^; (**b**) CV curves of CoVO-HNC at different scan rates from 5–50 mV s^−1^; (**c**) specific capacitance of CoVO-HNC at different current densities; (**d**) GCD curve comparison of CoO-HNC, CoVO-HNC, and CoVO-NP at 1 A g^−1^; (**e**) GCD curves of CoVO-HNC at different current densities from 1–10 A g^−1^; (**f**) cycling performance of CoVO-HNC at 10 A g^−1^; (**g**) relationship between log(*i*) and log(*v*) plots at corresponding anodic and cathodic peaks; (**h**) Q*_v_* versus *v^−1^*^/*2*^ and (**i**) the plot of 1/Q*_v_* versus *v^1^*^/*2*^ for the CoVO-HNC electrodes in 3M KOH electrolyte; (**j**)the ratio of the charge storage contribution (surface capacitive and diffusive) for the CoVO-HNC electrodes; (**k**) CV graph indicating the contribution of surface-controlled process for the charge storage of the CoVO-HNC at 5 mV s^−1^; (**l**) contribution rate generated by the surface capacitive and diffusion controlled process at various scanning speeds.

The samples were electrochemically tested using a three-electrode system. Figure 4a shows the CV curves of CoO-HNC, CoVO-HNC, and CoVO-NP at a scan rate of 5 mV s^−1^. The presence of significant redox peaks with pseudocapacitance properties for all samples in the potential range 0–0.6 V indicates that the charge storage originated from the redox reaction between cobalt ion and hydroxyl [36]. Furthermore, the integral area of CoVO-HNC is larger than that of CoO-HNC and CoVO-NP, indicating that CoVO-HNC has a stronger charge storage capacity. This is mainly attributed to the large specific surface area and mesoporous structure, as well as the enhanced conductivity due to the synergistic effect of cobalt and vanadium. The CV curves of CoO-HNC and CoVO-NP at a scan rate of 5–50 mV s^−1^ are presented in Figure S3a,c. Figure 4b exhibits the CV curves of CoVO-HNC at different scan rates from 5–50 mV s^−1^. As the scan rate increases, the integral area of the CV curve increases, while the shape of the CV curve changes slightly. This is because the active material does not have enough time to fully react with the electrolyte, and the utilization rate of the active material decreases [37]. Furthermore, the active material polarization effect becomes apparent as the scan rate increases [38]. The oxidation peaks at 0.15–0.2V and the reduction peaks at 0–0.5V can be clearly seen in the CV curves. These redox peaks are caused by the Faradaic reaction due to the electron transfer of CoVO in the KOH electrolyte [39]. The possible chemical reactions are as follows [40]:

$${\mathrm{Co}}^{2+}+3\;{\mathrm{OH}}^{-}↔\mathrm{CoOOH}+H_{2}O+e^{-}$$

$$\mathrm{CoOOH}+{\mathrm{OH}}^{-}↔{\mathrm{CoO}}_{2}+H_{2}O+e^{-}$$

Figure S4 shows the Nyquist plots in the open circuit voltage. The intercept on the real axis represents the equivalent series resistance (R_s_), including intrinsic resistance, interface contact resistance, and electrolyte resistance [41]. The diameter of the semicircle represents the charge transfer resistance (Rct), which reflects the charge transfer efficiency at the interface between the electrode and the electrolyte [42]. Meanwhile, the slope of the straight line in the low frequency region is the Warburg resistance, which represents the diffusion resistance of ions in the electrode material [43]. After fitting the equivalent circuit diagram (the inset in Figure S4), it was found that the R_s_ of CoVO-HNC (0.52 Ω) is lower than that of both CoO-HNC (0.59 Ω) and CoVO-NP (0.71 Ω). The R_ct_ of CoVO-HNC (0.22 Ω) is also lower than that of both CoO-HNC (0.54 Ω) and CoVO-NP (0.25 Ω). This implies that the CoVO-HNC has better conductivity and charge transfer rate. Compared with the other two samples, CoVO-HNC has the largest linear slope in the low frequency region. This suggests that it can achieve lower diffusion resistance and shorter ion diffusion path during the Faradaic reaction, which improves the diffusion and transport of electrolyte ions in the electrode material (Faradiac reaction—redox reactions caused by electron transfer. Diffusion—the movement of particles under the action of chemical potential gradient.) [44].

Figure 4d reveals the GCD curves of CoO-HNC, CoVO-HNC, and CoVO-NP at 1 A g^−1^ current density. The typical charge-discharge plateaus of these GCD curves further demonstrate the pseudocapacitive properties. However, the discharge time of CoVO-HNC is much longer, indicating a larger specific capacitance. The specific capacitances of CoO-HNC, CoVO-HNC, and CoVO-NP are calculated to be 254.22, 427.64, and 335.02 F g^−1^, respectively. For comparison, the GCD curves of CoO-HNC and CoVO-NP at 1–10 A g^−1^ are exhibited in Figure S3b,d. In Figure 4e, the GCD curve of CoVO-HNC at a current density of 1–10 A g^−1^ shows a high degree of symmetry and no significant IR drop. This demonstrates that CoVO-HNC has excellent reversibility and electrical conductivity of the material. As shown in Figure 4c, the specific capacitance values of CoVO-HNC were 427.64, 421.45, 416.21, 405.78, and 379.89 F g^−1^ at current densities of 1, 2, 3, 5, and 10 A g^−1^, respectively. The CoVO-HNC exhibits excellent rate performance with a capacity retention of 88% at a high current density of 10 A g^−1^. As seen in Figure 4f, CoVO-HNC retains 89.38% of the initial specific capacitance and the Coulombic efficiency is close to 100% after 10,000 charge-discharge cycles at a current density of 10Ag^−1^. This demonstrates the excellent cycling stability of CoVO-HNC as a supercapacitor electrode material. The excellent electrochemical ability of CoVO-HNC can be attributed to the following: (1) As the BET experiment confirmed, the nanocubic structure has a large specific surface area compared to nanoparticles. The large specific surface area provides more electroactive sites, facilitating the contact between the electrode material and electrolyte ions; (2) the mesoporous hollow nanocubes can provide transport pathways for ion/electron diffusion; (3) the hollow structure can effectively alleviate the volume change during the long charge and discharge process and prevent the structure from collapsing.

In order to clearly reveal the charge storage mechanism of CoVO-HNC electrode materials, it is of great significance to study their electrochemical kinetics [45]. The peak current (*i*) versus scan rate (*v*) in the CV curve obey the following relationship [46]:

(5) $$i=a\;v^{b}\;$$

Among them, *a* and *b* are two variable parameters. According to Equation (5), the value of *b* can be determined by the fitted slopes of log(*i*) and log(*v*). It is worth noting that when the b value is close to 0.5, the electrochemical process is dominated by diffusion, and when the b value is close to 1, the surface capacitive behavior is dominated [47]. As can be seen in Figure 4g, the *b* values of the CoVO-HNC anode and cathode peaks are 0.84 and 0.86, respectively. From this result, it can be inferred that the surface capacitive behavior contributes the most to the capacity. In addition, the contribution of surface capacitive behavior in charge storage was calculated using Trasatti’s analysis method [48,49]. Figure 4h shows a plot of Q*_v_* (Q*_v_* is the total measured voltammetric charge) versus *v^−1^*^/*2*^ (*v* is the scan rate). When *v* approaches infinity, the linear equation can be fitted as $Q_{v}=194.91+143.99v^{-1/2}$. The intercept represents surface capacitive charge storage and has a value of 194.91 C g^−1^. Figure 4i shows the relationship between 1/Q*_v_* and *v^1^*^/*2*^. After linear fitting, the equation $1/Q_{v}=0.00328+2.61v^{1/2}$ is obtained, and the intercept is the reciprocal of the total stored charge. When *v* approaches zero, the total stored charge is 304.87 C g^−1^. After calculation, it can be concluded that the value of diffusion charge storage is 109.96 C g^−1^. It can be clearly seen from Figure 4j that the surface capacitive charge storage accounts for 60% of the total charge storage, while the diffusion charge storage accounts for 40%. Apparently, the surface capacitive charge storage dominates throughout the electrochemical process.

The proportion of the contribution of diffusion and surface capacitive at different scan rates can be calculated by the following formula [46]:

(6) $$i\left(v\right)=k_{1}v+k_{2}v^{1/2}\;$$

Among them, $k_{1}$ and $k_{2}$ are two constant parameters, $k_{1}v$ represents the contribution ratio derived from the surface capacitive behavior, and the second half $k_{2}\;v^{1/2}$ represents the contribution determined by the diffusion-induced insertion process. As shown in Figure 4h, the surface capacitance contribution is marked by the blue shaded region in CV curve at 5 mV s^−1^. Figure 4l presents the histogram of surface capacitance and diffusion contribution at different scanning rates. The surface capacitive contributions of the CVO-HNC are 64.4%, 67.7%, 74.1%, 80.7%, and 94.3% when the scan rates are 5, 10, 20, 30, and 50 mV s^−1^, respectively. Apparently, the surface capacitive behavior dominates throughout the electrochemical process. However, the contribution of diffusion control decreases with the increase of scan rate because there is almost no time for the diffusion control process at a high scan rate [50].

**Figure 5:**
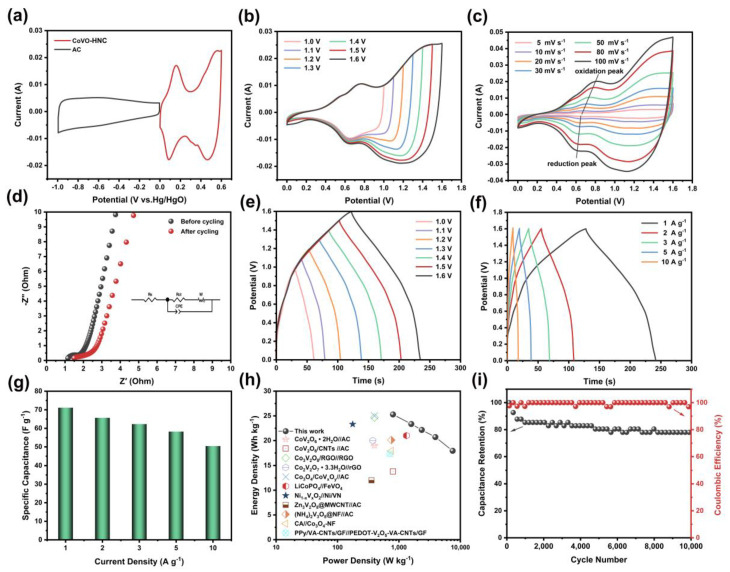
(**a**) CV curves of AC and CoVO-HNC electrodes at a scan rate of 10 mV s^−1^; (**b**) CV curves of CoVO-HNC//AC at different potential windows at a scan rate of 50 mV s^−1^; (**c**) CV curves of CoVO-HNC//AC at different scan rates from 5–100 mV s^−1^; (**d**) Nyquist plot of CoVO-HNC//AC; (**e**) GCD curves of CoVO-HNC//AC in different voltage windows at 1 A g^−1^; (**f**) GCD curves of CoVO-HNC//AC at diverse different current densities; (**g**) specific capacitances at different current densities; (**h**) Ragone plots of the CoVO-HNC//AC device and the comparison of other reported ASCs devices; (**i**) cycling performance of CoVO-HNC//AC at 10 A g^−1^.

To evaluate the practical application of CoVO-HNC, a CoVO-HNC//AC ASC was assembled using CoVO-HNC and AC as electrodes. The CV curve of CoVO-HNC and AC was measured at a scan rate of 10 mV s^−1^ as shown in Figure 5a, and the operation voltage windows were 0–0.6 V and −1.0–0 V, respectively. Figure 5b shows the CV curves of CoVO-HNC//AC at various upper cutoff potentials at 50 mV s^−1^. The cutoff potential of CoVO-HNC//AC ASC was clearly found to be 1.6 V without obvious oxygen evolution reaction. Figure 5c shows the CV curve of CoVO-HNC//AC at different scan rates. It can be clearly found that a pair of redox peaks belong to a typical Faraday redox reaction. Furthermore, the curve shape can still be well maintained with the increase of the scan rate, indicating that the CoVO-HNC//AC has excellent reversibility. The Nyquist impedance spectrum of the CoVO-HNC//AC is shown in Figure 5d. The initial Rs was 1.21 Ω, and after 10,000 cycles the Rs became 1.49 Ω. It can be found that the value of Rs changes little before and after cycling, indicating that CoVO-HNC//AC has good electrical conductivity. The values of Rct before and after cycling are 0.53 Ω and 0.94 Ω, respectively, which means that its charge transfer rate decreases after charge-discharge cycles. A near vertical line is observed in the low frequency region, which indicates the quasi-ideal capacitive behavior of the device. Figure 5e represents the GCD curves at the same current density under different voltage windows (1.0–1.6 V). Consistent with the results in Figure 5b, it is demonstrated that the operating voltage window of CoVO-HNC//AC is 1.6V. In order to further test the energy storage performance, GCD tests were carried out at different current densities, as shown in Figure 5f. The nearly symmetrical triangular feature of the GCD curve at various current densities reveals its excellent reversibility and high Coulomb efficiency [51]. The specific capacitance of CoVO-HNC//AC was 71.11, 65.65, 62.29, 58.23, and 50.43 F g^−1^ at current densities of 1, 2, 3, 5, and 10 A g^−1^, respectively (Figure 5g). Obviously, even at a current density of 10 A g^−1^, 70.9% of the original capacitance is still retained, which proves the excellent rate performance of the ASC. As shown in the Ragone plots of Figure 5h, the assembled ASC shows a high energy density of 25.28 Wh kg^−1^ at a power density of 801.24 W kg^−1^ and can still maintain an energy density of 17.93 Wh kg^−1^ even at a power density of 7505.86 W kg^−1^. Its energy density and power density are significantly higher than other works, see Table 1. Figure 5i demonstrates the cycle performance of the CoVO-HNC//AC at a current density of 10 A g^−1^. After 10,000 charge-discharge cycles, CoVO-HNC//AC exhibited excellent cycling stability with a capacitance retention of 78% and a Coulombic efficiency close to 100%. In consideration of the above results, the CoVO-HNC electrode material has excellent supercapacitor performance and the assembled CoVO-HNC//AC ASC device has practical application prospects.

## 4. Conclusions

In this study, CoVO-HNC was prepared by a solvothermal method using ZIF-67 as a template. CoVO-HNC with a large specific surface area provides more electroactive sites, facilitating the contact between the electrode material and electrolyte ions. At the same time, the hollow and mesoporous structure shortens the electron transfer path during the electro-chemical reaction. Thanks to this structure, the CoVO-HNC has a specific capacitance of 427.64 F g^−1^ at a current density of 1 A g^−1^. In addition, when the power density is 801.24 W kg^−1^, the energy density is 25.28 Wh kg^−1^ and still retains 17.93 Wh kg^−1^ at a high-power density of 7505.86 W kg^−1^. The hollow structure effectively alleviates the structural collapse caused by the volume expansion during the charging and discharging process, thereby increasing the service life and cycling stability. Therefore, the capacitance retention rate of CoVO-HNC//AC is 78% after 10,000 charge-discharge cycles at a current density of 10 A g^−1^, and its Coulombic efficiency is close to 100%. Such excellent electrochemical performance suggests that CoVO-HNC has huge application potential in supercapacitors.

## Figures and Tables

**Table 1:** Comparison of electrochemical performance of the assembled Co-VO-HNC//AC ASC with ASC of similar materials.

ACS	Specific Capacitance	Cycling Performance	Power Density	Energy Density	Ref.
CoVO-HNC//AC	71.11 F g^−1^ (at 1 A g^−1^)	78% (after 10,000 cycles at 10 A g^−1^)	801.24 W kg^−1^	25.28 Wh kg^−1^	This work
Co_2_V_2_O_7_·3.3H_2_O//rGO	60.7 F g^−1^ (at 1 A g^−1^)	96.4% (after 10,000 cycles at 3 A g^−1^)	375.8 W kg^−1^	19 Wh kg^−1^	[13]
CoV_2_O_6_·2H_2_O//AC	47.2 F g^−1^ (at 1 A g^−1^)	78.6% (after 35,000 cycles at 5 A g^−1^)	400 W kg^−1^	19 Wh kg^−1^	[14]
PPy/VA-CNTs/GF//PEDOT-V_2_O_5_-VA-CNTs/GF	48.83 F g^−1^ (at 1 A g^−1^)	64% (after 5000 cycles at 10 A g^−1^)	710	17.34 Wh kg^−1^	[52]
Zn_3_V_2_O_8_@MWCNT//AC	41.2 F g^−1^ (at 1 A g^−1^)	81% (after 5000 cycles at 5 A g^−1^)	358 W kg^−1^	12 Wh kg^−1^	[53]
CoV_2_O_6_/CNTs//AC	38.7 F g^−1^ (at 1 A g^−1^)	88% (after 9000 cycles at 1 A g^−1^)	800 W kg^−1^	13.8 Wh kg^−1^	[54]
CA//Co_3_O_4_-NF	57.4 F g^−1^ (at 1 A g^−1^)	85% (after 1000 cycles at 2 A g^−1^)	750	17.9 Wh kg^−1^	[55]
Co_3_V_2_O_8_/RGO//RGO	127.62 C g^−1^(at 0.5 A g^−1^)	91.64% (after 10,000 cycles at 5 A g^−1^)	400 W kg^−1^	24.66 Wh kg^−1^	[56]
Co_3_O_4_/CoV_x_O_y_//AC	66.5 F g^−1^ (at 1 A g^−1^)	83.4% (after 5000 cycles at 10 A g^−1^)	400 W kg^−1^	25 Wh kg^−1^	[57]
Ni_1-x_V_x_O_2_//Ni/VN	65.7 F g^−1^ (at 2.5 mA cm^−1^)	87% (after 1000 cycles at 10 mA cm^−1^)	176.7 W kg^−1^	23.3 Wh kg^−1^	[58]
LiCoPO_4_//FeVO_4_	73 F g^−1^ (at 3 mA cm^−1^)	97.3% (after 1000 cycles 5 mA cm^−2^)	1326 W kg^−1^	21 Wh kg^−1^	[15]
(NH_4_)_2_V_3_O_8_@NF//AC	66.2 F g^−1^ (at 1 A g^−1^)	61.5% (after 5000 cycles at 2 A g^−1^)	752.0 W kg^−1^	20.1 Wh kg^−1^	[59]

## Data Availability

Not applicable.

## Supplementary Materials

The following supporting information can be downloaded at: https://www.mdpi.com/article/10.3390/nano12050848/s1, Figure S1: XRD pattern of ZIF-67, Figure S2: Nitrogen absorption/desorption isothermal and pore size distribution curves of (a) CoVO-HNC, (b) CoVO-NP, Figure S3: (a) CV curves of CoO-HNC at scan rates of 5–50 mV s^−1^; (b) GCD curves of CoO-HNC at current densities of 1–10 A g^−1^; (c) CV curves of CoVO-NP at scan rates of 5–50 mV s^−1^; (d) GCD curves of CoVO-NP at current densities of 1–10 A g^−1^. Figure S4: Nyquist plots of CoO-HNC, CoVO-HNC, and CoVO-NP.
