# Supplementary material for: ZIF-67 Derived Co2VO4 Hollow Nanocubes for High Performance Asymmetric Supercapacitors
Source: Nanomaterials (Basel). 2022 Mar 2;12(5):848. doi: 10.3390/nano12050848 (PMC8912880; doi:10.3390/nano12050848)
Supplement: Supplementary file 1 [file nanomaterials-12-00848-s001.zip › nanomaterials-1604375-supplementary.pdf]

nanomaterials

Supporting Information

ZIF-67 Derived Co_2VO_4 Hollow Nanocubes for High Performance Asymmetric Supercapacitors

Chengda Li [1,2], Dongliang Ma [1,2,*] and Qinglin Zhu [1,2]

[1] Xinjiang Key Laboratory of Solid State Physics and Devices, Xinjiang University, Urumqi 830017, China; lichengda572@163.com (C.L.); linzyznil@163.com (Q.Z.)
[2] School of Physical Science and Technology, Xinjiang University, Urumqi 830017, China
* Correspondence: dlma@xju.edu.cn

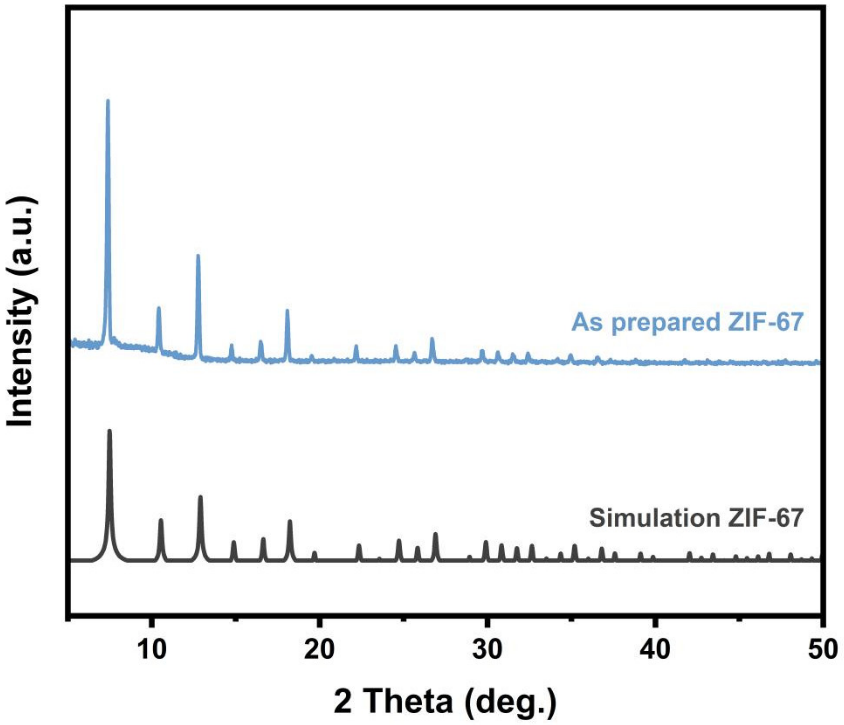

Figure S1. XRD pattern of ZIF-67.

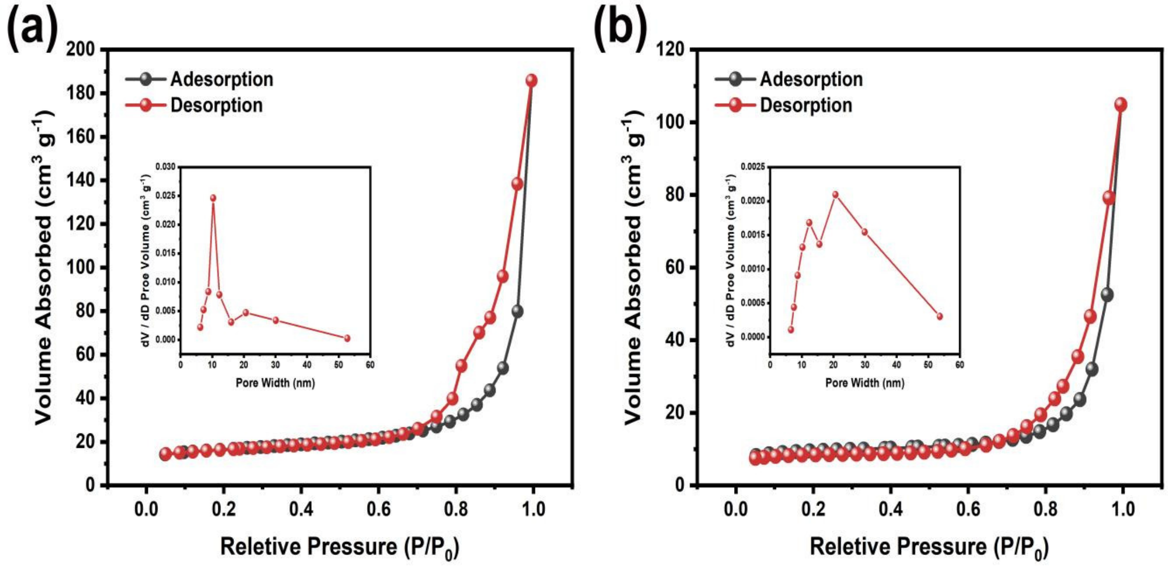

Figure S2. Nitrogen absorption/desorption isothermal and pore size distribution curves of (**a**) CoVO-HNC, (**b**) CoVO-NP.

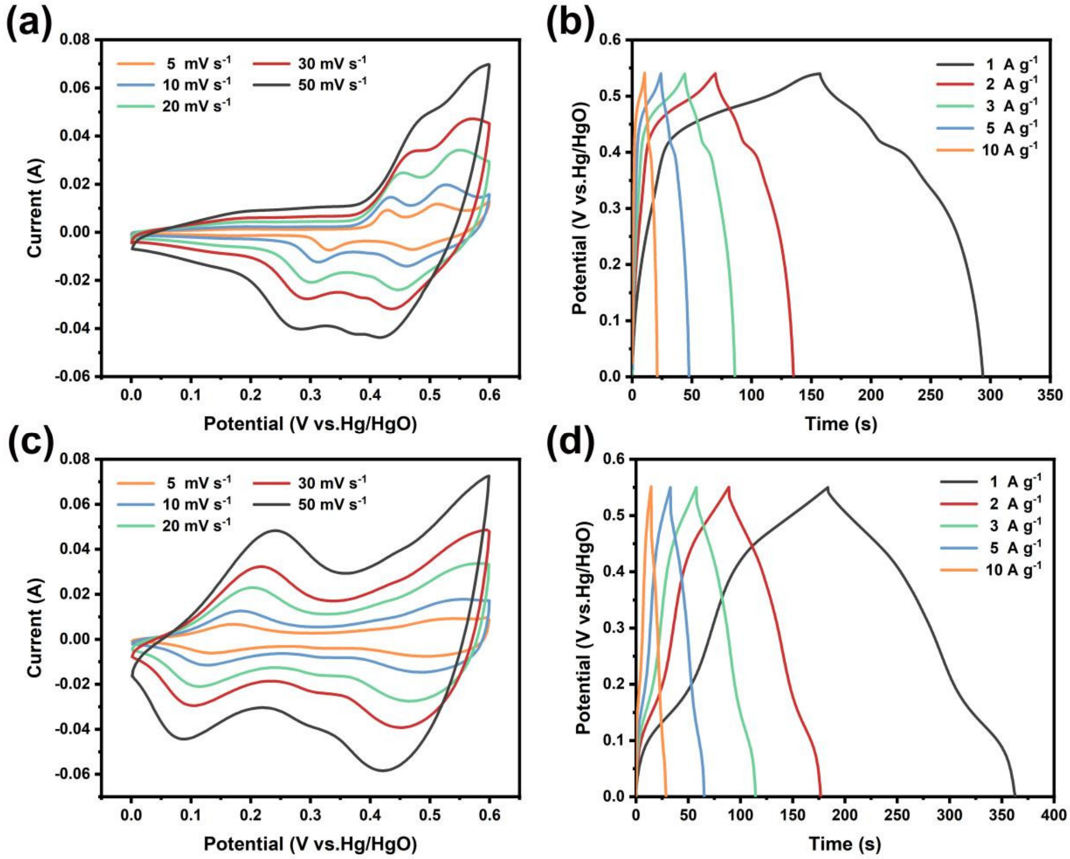

Figure S3. (**a**) CV curves of CoO-HNC at scan rates of 5-50 mV s^{-1}; (**b**) GCD curves of CoO-HNC at current densities of 1-10 A g^{-1}; (**c**) CV curves of CoVO-NP at scan rates of 5-50 mV s^{-1};(**d**) GCD curves of CoVO-NP at current densities of 1-10 A g^{-1}.

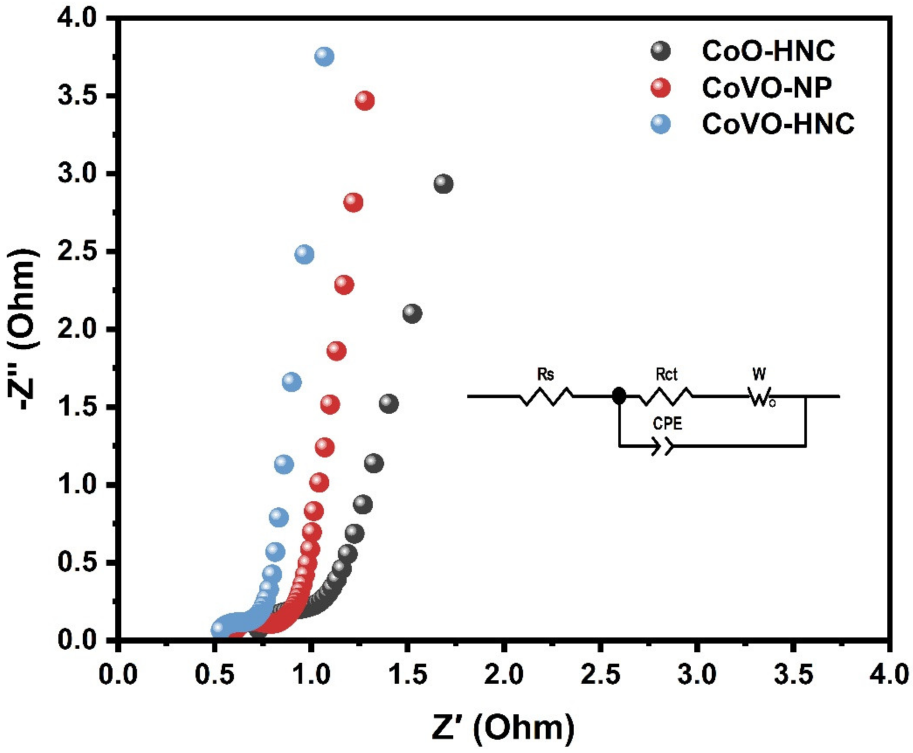

Figure S4. Nyquist plots of CoO-HNC, CoVO-HNC and CoVO-NP .
